# Individual cerebrocerebellar functional network analysis decoding symptomatologic dynamics of postoperative cerebellar mutism syndrome

**DOI:** 10.1093/texcom/tgac008

**Published:** 2022-02-11

**Authors:** Ko-Ting Chen, Tsung-Ying Ho, Tiing-Yee Siow, Yu-Chiang Yeh, Sheng-Yao Huang

**Affiliations:** Department of Neurosurgery, Chang Gung Memorial Hospital at Linkou, Taoyuan 333, Taiwan; School of Medicine, Chang Gung University, Taoyuan 33302, Taiwan; Neuroscience Research Center, Chang Gung Memorial Hospital at Linkou, Taoyuan 333, Taiwan; Department of Nuclear Medicine and Molecular Imaging Center, Chang Gung Memorial Hospital at Linkou, Chang Gung University, Taoyuan 333, Taiwan; Department of Medical Imaging and Intervention, Chang Gung Memorial Hospital at Linkou, Taoyuan 333, Taiwan; Department of Neurosurgery, Chang Gung Memorial Hospital at Linkou, Taoyuan 333, Taiwan; Molecular Medicine Research Center, Chang Gung University, Taoyuan 33302, Taiwan

**Keywords:** cerebellar mutism syndrome, connectome, functional connectivity, network neuroscience, posterior fossa syndrome

## Abstract

**Introduction:**

Postoperative cerebellar mutism syndrome (pCMS) consists of three types of symptoms (motoric, linguistic, and neurobehavioral) in patients with posterior fossa pathologies. The evolutional mechanism of this high cognitive syndromic complex from cerebellar origin remains unconfirmed. Previous studies analyzing CMS patients mostly focused on the association between structural abnormalities that occur during CMS, of which proximal efferent cerebellar pathway (pECP) injury appears to be the most common pathogenesis. However, structural imaging may not be sensitive enough to determine the dynamic course of CMS, since the symptomatology is primarily an output of cerebral operation.

**Method:**

We took a network approach in a child during her course of development and recovery of the pCMS. On the other hand, a network neuroscience approach using a mathematical model to extract information from functional imaging to generate interregional connectivity provides abundant evidence that the cerebellum is influential in modulating cerebral functions.

**Result:**

This study applied a network approach to children with pCMS. An individual cerebrocerebellar functional network analysis using graph theory was then performed to determine the network dynamics during CMS. Cross-validation of clinical neurophysiology and functional neuroscience suggested the critical role of the pECP within CMS from the network analysis.

**Conclusion:**

The employed approach was therefore useful in determining the complex clinical symptoms using individual functional network analysis, which bridges the gap between structural neuroimaging and clinical neurophysiology.

## Introduction

Cerebellar mutism (CM) refers to muteness following lesions to the cerebellum as opposed to the cerebrum or lower cranial nerves (Gudrunardottir et al. 2011). A broad aspect of clinical presentations including cognitive, affective, and neurological symptoms has been described in addition to speech disorder since the first description of akinetic mutism after resecting a cerebellar tumor in a child (Daly and Love 1958; Riva 1998; Riva and Giorgi 2000). Thereafter, cerebellar mutism syndrome (CMS) and, later, posterior fossa syndrome (PFS; Kirk et al. 1995) have been used to describe patients of such complex set of clinical signs and symptoms. Three categories of symptomatology have been proposed, which include linguistic, motoric, and neurobehavioral (Gudrunardottir et al. 2011), of which mutism, hypotonia, and emotional irritability being the triad of CMS, while the PFS is recognized as a broader term to encompass CMS (Gudrunardottir et al. 2011; Catsman-Berrevoets and Patay 2018).

Although the syndrome has been discovered for decades, the pathophysiology is not fully understood. Researchers have applied various methodologies to approach different aspects of CMS or PFS, such as neuropsychological examination to measure clinical psycho-behavioral domains (Mariën et al. 2013), signal change on anatomical specific regions in magnetic cerebral images (magnetic resonance imaging [MRI]) (Sergeant et al. 2017), diffusion tensor imaging (DTI) to quantify structural integrity of responsible fiber tracts (Morris et al. 2009; Palesi et al. 2015; Avula 2020), and single photon emission computed tomography or positron emission tomography (PET) to detect perfusion or metabolic change of specific cerebral regions (Erşahin et al. 1996; Sagiuchi et al. 2001; Miller et al. 2010). Collectively, proximal efferent cerebellar pathway (pECP) appeared to play a critical role in the pathophysiology to a broad clinical symptomatology from CMS to PFS (Koh et al. 1997; Morris et al. 2009). The cerebellar function beyond motor learning and control has long been recognized (Leiner et al. 1987). Primarily, cerebellum serves as a modulator to optimize cerebral cortical output signals through intensive reciprocal cerebellocerebral connection, mainly the pECPs (Leiner et al. 1987). The pECP arises from dentate nuclei, which give fibers to superior cerebellar peduncle (SCP), decussate in the mesencephalic tegmentum, and synapse in the ventral lateral and ventral anterior nuclei of the thalamus (Trobe 2010). The corresponding postsynaptic neurons project to widespread cortical areas, including prefrontal, premotor, primary motor, primary sensory, parietal association, temporal, and limbic cortices (Catsman-Berrevoets and Aarsen 2010; Miller et al. 2010; Trobe 2010; Mariën et al. 2013). Such pan-cerebral cortical connections suggesting global function depletion to varies extent once pECP projection fibers are violated. The theory is further supported by the cerebellar cognitive affective syndrome proposed by Schmahmann et al. in adult population following cerebellar stroke, infection, or atrophy (Schmahmann 2019).

Few evidence are available to elucidate the relationship between the violation of pECP and diverse symptomatologies. The first reason is the wide diversity of presentation of symptoms within each category of symptomatology (Gudrunardottir et al. 2011a) and the second one is the various degree of severity of each symptom (Robertson et al. 2006). Such limitation makes a groupwise analysis being confined to syndromic rather than symptomatologic study, which further limits a closer examination of CMS from neural functional prospects. Besides, the dynamic in variety and severity of CMS requires modalities to interrogate functional rather than structural aspect of both cerebellar and cerebral regions (Toescu et al. 2018). To analyze these complex issues for further understanding the CMS, we took time domain as the *X* axis and the severity of symptoms as *Y* axis to display the dynamic of symptomatology, and in the meanwhile, a serial PET studies were undertaken at the initial and recovered stages of the CMS in a pediatric patient who underwent surgical resection for a fourth ventricular medulloblastoma. An individual functional network analysis was used to study the cerebrocerebellar functional connectivity (FC) in an intra- and inter-staged fashion. We aim to provide insights into the pathomechanism of CMS and to introduce a novel approach as an opportunity to unravel mysteries of complex and dynamic neurological symptoms/syndromes for pathologies in the CNS.

## Material and methods

### Categories and grading of postoperative CMS

**Fig. 1:**
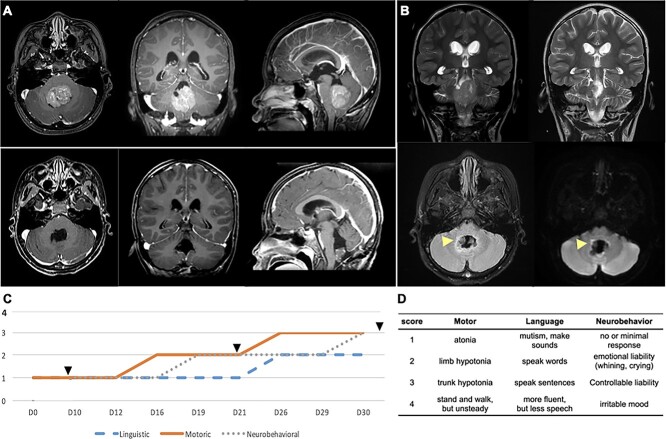
Surgical resection and postoperative course of pCMS. A) The pre- and postoperative MRI showed complete resection of medulloblastoma located at fourth ventricle via a midline trans-lower vermian approach. B) A small high signal area (white arrowhead) noted on the postoperative coronal T2-weighted imaging, axial T2 FLAIR, and DWI sequence, indicating an edema of SCP. C) A time scale of the dynamic improvement of 3 domains of symptoms for pCMS was recording according to a scoring system modified from Gudrunardottir et al.,^1^ which categorized the symptomatology of pCMS into motoric, linguistic, and neurobehavioral domains. PET studies have been arranged along this time course (black arrowhead). A score of 1–4 is graded according to the severity of symptoms within each category (D). (High-resolution figure in Supplementary Files.)

The pre- and postoperative MRI was demonstrated to illustrate signal change along the dentate nucleus (DN) and the SCP (proximal pECP, Fig. 1A and B). The PFS developed since postoperative day (POD) 2, which became progressively prominent and recovered nearly totally within 3 months. The symptomatology was categorized into motoric, linguistic, and neurobehavioral domains according to Gudrunardottir et al. (2011b). The timeline of recovery of these 3 domains of symptoms as well as 3 of 4 ^18^F-fluorodeoxyglucose (^18^FDG)-PET studies was marked on the plot (Fig. 1C). A scoring system was designed according to the severity of symptoms within each category. In motoric domain: atonia, limb hypotonia, trunk hypotonia, and able to stand and walk belongs to score 1, 2, 3, and 4, respectively. In linguistic domain: mutism, speak words, sentence, and fluent speech belongs to score 1, 2, 3 and 4, respectively. In neurobehavioral domain: minimal response, emotional liability, liability but controllable and irritable mood belongs to score 1, 2, 3, and 4, respectively (Fig. 1D).

### Metabolic imaging studies

The Institutional Review Board has approved the project (Chang Gung Medical Foundation Institution Review Board No. 202101309B0), and we have a full consent that has been acquired from her guardian before each study and data analysis. The patient was required to fast for at least 4 h before injection of ^18^F-FDG. ^18^F-FDG-PET scans were acquired using a Biograph mCT PET/computed tomography (CT) (Siemens Medical Solutions, Malvern, PA) or Discovery MI PET/CT (GE Healthcare, Milwaukee, WI) 30 min after injection. The 3-D OSEM (ordered subset expectation maximization) PET reconstruction algorithm (4 iterations, 24 subsets; Gaussian filter: 5 mm) with CT-based attenuation correction was applied to obtain PET images of a matrix size of 400 × 400. We perform daily quality control before any scan begins in order to make sure that the value of ECF behavior (Emission Calibration Factor, per Siemens terminology) between these 2 scanners is within 3%. A total of 4 separate PET studies was applied (Fig. 1C). We assign stages 1, 2, 3, and 4 to represent each timepoint of PET study, and therefore, several correlation coefficient matrices could be generated by comparing 2 chosen stages as shown in Supplementary Fig. S1.

### Individual metabolic brain network

#### Image analysis

All downloaded PET data were processed using PMOD image analysis software (version 3.3; PMOD Technologies Ltd, Zurich, Switzerland) and spatially normalized into the Automated Anatomical Labeling (AAL) space. All images were automatically segmented into 116 anatomical structures (volumes of interest) using the AAL atlas (He et al. 2009). For the standard quantification procedure of the FDG image, the regional radioactivity concentration was first converted to standardized uptake values (SUVs) (Lucignani et al. 2004). Then, the regional SUV ratio (SUVR) of the mean SUV between the target and reference regions was calculated with the pons as the reference region. Finally, each subject’s regional SUVR for each AAL structure was extracted to construct the SUVR data matrix.

#### Network SUVR differences

**Fig. 2:**
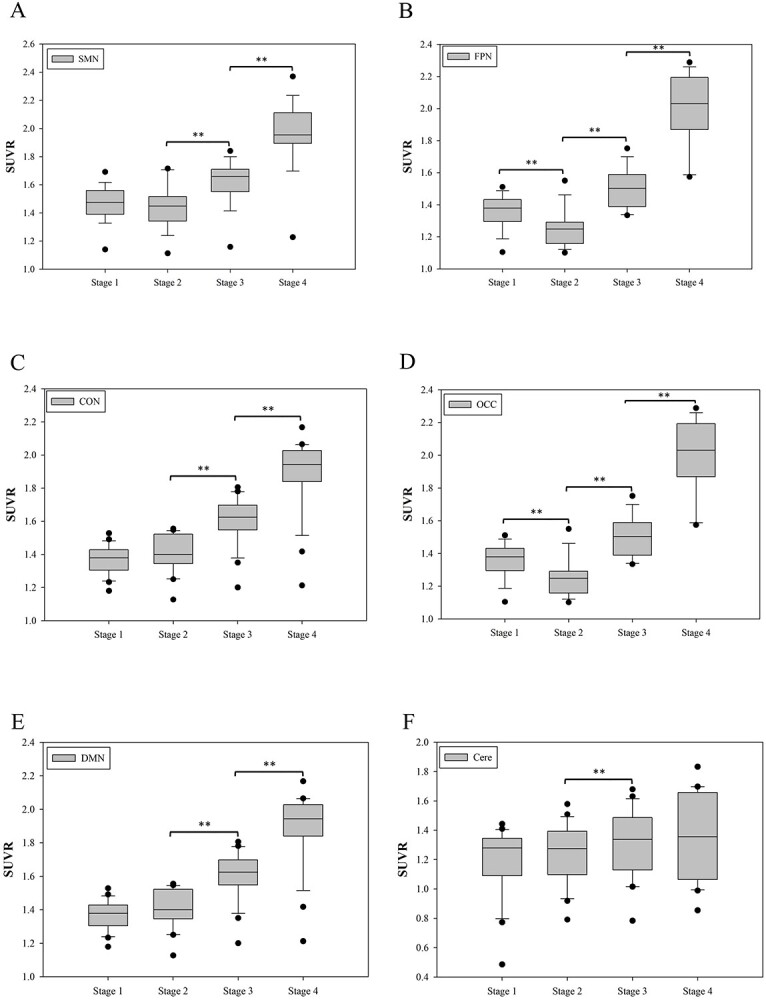
Metabolic dynamics of 6 functional networks in 4 timepoints (stages 1, 2, 3, and 4 represent POD 7, 21, 36, and 93). Among the 6 networks, the SMN (A), CON (C), and DMN (E) were the 3 networks got earlier improvement and kept improving across all timepoints. A significant decrease of FPN (B) and OCC (D) at stage 2 comparing to stage 1. The cerebellar network (F) was relatively stable across all timepoints except a mild yet significant uptake increases from stage 2 to 3 (one-way analysis of variance with post hoc Tukey’s multiple comparisons; Cere, cerebellar network).

Figure 2 shows that the SUVR data matrix was then applied to calculate a significant SUVR for each subnetwork in different time points. Six subnetworks were constructed for further analysis including sensorimotor network (SMN), occipital network (OCC: calcarine, cuneus, occipital, lingual, and fusiform gyrus), fronto-parietal network (FPN), default mode network (DMN), cingulo-opercular network (CON), and cerebellum (26 regions) (He et al. 2009; Huang et al. 2020). We used one-way ANOVA analysis of variance with post hoc Tukey's multiple comparisons as specified in the figure legend (the differences are considered statistically significant if *P* < 0.05 ^*^ and *P* < 0.01 ^*^^*^). We modified Huang’s method for this study to calculate the changes of metabolic brain networks by ^18^FDG-PET image.

An effect size (ES) has been used to measure the amount of association between 2 variables or differences between 2 groups in an experiment (Becker 1988; Morris and DeShon 2002; Lucignani et al. 2004). We therefore used the ES method to calculate the difference of regional SUVR between the first and other timepoints after surgery. The individual brain network can be modified from the treatment effect measured from an independent timepoint (1st-2nd, 1st-3rd, 1st-4th) to calculate the regional difference between the single timepoint-subject SUVR deviation from the mean SUVR value of first subject in 2 regions (Huang et al. 2020).

$$ \mathrm{ESd}\left(i,j\right)=\frac{{\bar{x}}_{k,i}-{\bar{x}}_{1^{st},i}}{s_i}-\frac{{\bar{x}}_{k,j}-{\bar{x}}_{1^{st},j}}{s_j} $$

$$ k={2}^{\mathrm{nd}},{3}^{\mathrm{rd}},{4}^{\mathrm{th}}\ \mathrm{time}\ \mathrm{points} $$

Here, ESd indicates the difference of ES between 2 times and its value is affected by the correlation between regions *i* and *j* (*i* and *j* are within 120 region of interests) (Becker 1988; Morris and DeShon 2002). Let ${\bar{x}}_{k,i}$ and ${\bar{x}}_{k,j}$ be mean regional SUVR for regions *i* and *j* (*i ≠ j*) from the second, third, and fourth time points; ${\bar{x}}_{1^{st},i}$ and ${\bar{x}}_{1^{st},j}$ the mean regional SUVR from the first time point; *s_i_* and *s_j_* the corresponding standard deviation of SUVR. In current study, as the approach widely used by other researchers (Sanabria-Diaz et al. 2013; Imai et al. 2020), we discuss concurrent metabolic increase between 2 time points. As has been raised by Chen et al. (2018), it is difficult to deal with negative correlation at the time of binarization. Therefore, to focus on the network related to the clinical recovery of postoperative cerebellar mutism syndrome (pCMS), we specifically interrogated concurrent increased metabolism in paired ROIs (${\bar{x}}_{k,i}-{\bar{x}}_{1^{st},i}>0, and\ {\bar{x}}_{k,j}-{\bar{x}}_{1^{st},j}>0$). The ESd(*i, j*) can be calculated for all pair of region of interests (*i*, *j*) to obtain a final ESd matrix (120 × 120) and ESd(*i, j*) is also a bivariate normal distribution. By viewing ESd(*i, j*) as *Z* score (Kim 2015) and applying the simple Fisher transformation, the correlation coefficient value *R(i, j)* between *i*-th and *j*-th regions can be derived as:

$$ R\left(i,j\right)=\frac{\exp \left(2\ast \mathrm{ESd}\left(i,j\right)\right)-1}{\exp \left(2\ast \mathrm{ESd}\left(i,j\right)\right)+1} $$

where *0 < R*(*i, j*) *<* 1*. R*(*i, j*) is the correlation coefficient between *i*-th and *j*-th regions of the subject. If the ${\bar{x}}_{k,i}$ and ${\bar{x}}_{k,j}$ be extreme value of SUVR variation between 2 regions infers a smaller ESd(*i, j*) value (mean that: ${\bar{x}}_{k,i}$ and ${\bar{x}}_{k,j}$ have consistent increase) and this leads to a higher single-subject regional correlation coefficient. However, the transformation formula will generate a smaller value for *R*(*i,j*). Thus, to adjust this, we then applied the *R’*(*i, j*) *=* 1*-R*(*i, j*) as the final value for the regional correlation coefficient between the other time points and the stage 1. *R′*(*i, j*) is the correlation coefficient between *i*-th and *j*-th regions of the stages 2, 3, and 4 to 1. The final individual correlation coefficient matrix (i.e. connectivity matrix) can now be computed as *R′*(*i, j*). For detailed information, please refer to our previous article (Huang et al. 2020). The algorithm of individual brain network analysis is summarized in Supplementary Fig. S1.

#### Brain network

From the network theory, a network (or graph) is a mathematical model representing a collection of nodes (or vertices) and edges (or connections) between pairs of nodes. In our study, a connection in a brain network is defined in terms of statistical associations between each pair of brain regions among the 116 anatomical structures (He et al. 2009). In addition, we added 4 anatomical structures (pons, left and right DN, and red nucleus [RN]). The statistical association was obtained by synchronized co-variations and measured by computing their correlation coefficient values, across examines. Hence, an interregional correlation coefficient (*N* × *N*, where *N* is the number of brain regions; here, *N* = 120) for the statistical connections was calculated using all pairs of anatomical structures. To obtain a binary connectivity network, a threshold is needed. Other studies used a range of sparsity degrees from 0.5 to 0.9 as correlation coefficient values thresholds, but this led to variable results (Huang et al. 2020). Here, various thresholds ranging from 0 to 1, in steps of 0.01, yielding a set of 101 values, were shown in Supplementary Fig. S2, and that illustrates the plots for number of edges versus correlation coefficient values ranging at each brain network (stage 2-1, stage 3-1, stage 4-1). In our research, we only choose one of the correlation coefficient values to show our result in the figures.

**Fig. 3:**
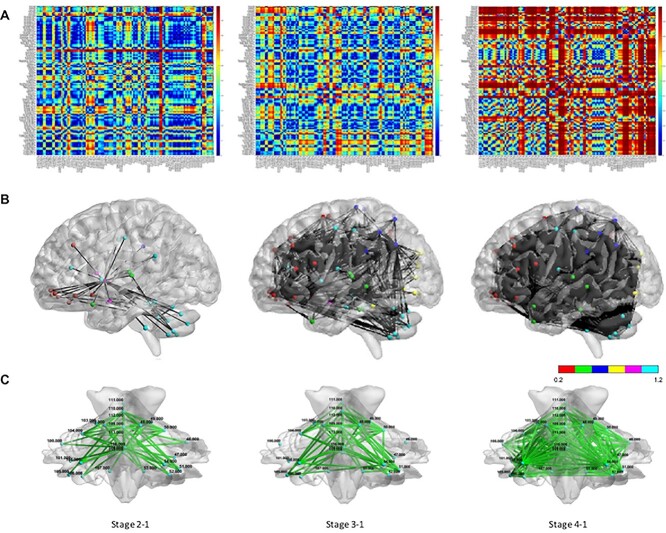
Metabolic correlation coefficient matrices and connectivity networks. To further analyze connectivity within each 116 anatomical structures of AAL atlas, metabolic correlation coefficient matrices were generated comparing stage 2, 3, and 4 to stage 1, respectively. A significantly and progressively enhanced connectivity was observed in correlation coefficient matrices (A) and connectivity networks of cerebrum (threshold: 0.95, B) and cerebellum (threshold: 0.65, C). (High-resolution figure in Supplementary Files.)

We further used the BrainNet Viewer (www.nitrc.org/projects/bnv/) toolbox to display connections forming the subnetwork. Then, graphs were generated comparing different timepoints of ^18^FDG-PET in stage 2-1, stage 3-1, and stage 4-1, respectively. As is seen in Fig. 3, the brain connectivity graphs were visualized for 3 types. The connections were indicated by black lines and nodes by the color dots (red for frontal lobe, green for temporal lobe, deep blue for parietal lobe, yellow for occipital lobe, pink for subcortical gray matter, light blue for other regions; Fig. 3) (Tzourio-Mazoyer et al. 2002).

## Results

### Clinical course of posterior fossa syndrome

A 9-year-old normal-developed girl presented with nausea, diplopia, and headache for 2 weeks. MRI showed a homogenous enhancing mass occupying fourth ventricle compressing brain stem and causing obstructive hydrocephalus (Fig. 1A). She underwent right frontal external ventricular drainage for emergent relieve of intracranial hypertension, and subsequently, a median suboccipital craniotomy trans-lower vermian approach was performed for tumor totally removal (Fig. 1A). The pathology reported medulloblastoma, classic, SHH-activated and *TP53*-wild type, world health organization grade 4.

**Fig. 4:**
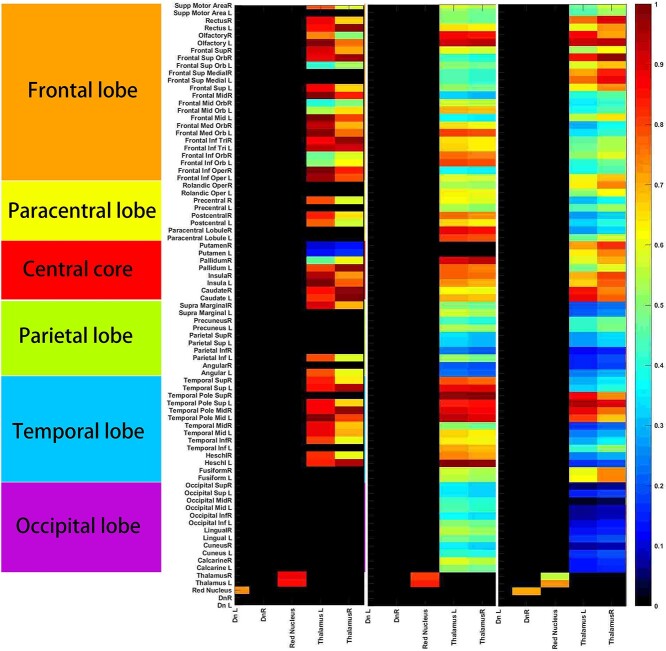
Continuous recovery of dentato-rubro-thalamo-cortical pathway using individualized connectivity network. To test the hypothesis of pECP injury as the pathophysiology of PFS, dentate nuclei, RN, and thalami were specifically selected (roots) to see the correlation between the seeds and the whole cerebral subregions (frontal, paracentral, central core, parietal, temporal, and occipital lobes). (High-resolution figure in Supplementary Files.)

Three hours after the operation, she was extubated and was able to obey order with her limbs moved freely and could drink water and even asked for food. Since postoperative 12 h, she became progressive drowsiness, mutism, and general atonia. MRI revealed complete tumor resection with increased signal of right DN and SCP in T2-weighted image and diffusion-weighted image, but no acute infarction (Fig. 1B). To rule out epileptic discharge, an electroencephalography was arranged but no focal abnormality was found. After implanting ventriculoperitoneal shunt on POD 6, she was transferred to ward with a condition of mutism, hypotonia, and whining. We performed the first PET imaging on POD 7 (stage 1, Figs 2–4), which revealed low global uptake and especially hypometabolism of deep cerebellar nucleus and bilateral frontal lobes. From POD 10 to 15, her time of arousal increased, and some involuntary eyeball or eyelid movement was noted. She had few movements of legs but not arms and emotional lability (e.g. crying, whining, or even screaming). We gave her dopamine agonist (Sinemet 25/100, Levodopa 100 mg + Carbidopa 25 mg, 0.5 tablet twice a day) since POD 13. From POD 16 to 20, she turned into frequent laughing to stimulus (pathological laughing) and the whining became less and more consolable. Her left hand started to move earlier than right hand, but there was still prominent truncal hypotonia including her neck. Besides, her eyes remained closed most of the time. The second PET on POD 21 (stage 2, Figs 2–4) revealed increase global metabolism, but a decrease in temporal–occipital and frontal cortices was more significant. On POD 26, a significant improvement of muscle tone and truncal stability was noted. She opened eyes at daytime and followed orders well. She tried to speak with different mouth shapes but there were no sounds except “aha” and laughing. From POD 28 to 34, she started to speak words, counting numbers, writing her name, and practicing standing. We removed her nasogastric tube on POD 30. The third PET on POD 36 (stage 3, Figs 2–4) reported improvement of global perfusion with resolving of previous cortical hypometabolic areas. Shortly after the third PET, she was discharged home with the ability to walk for a short distance. Her Romberg test was normal, and there was mild dysmetria on the finger-nose-finger test. The timeline of symptomatology of PFS is shown in Fig. 1C and D. The fourth PET that was done on POD 93 (stage 4, Figs 2–4) showed a further improvement of global metabolism. During that time, she was orientated, can walk independently but cannot run due to trunk unsteadiness, still less willing to speak but could communicate. Her mood was irritable, and she had difficulties to control her temper. She could attend class in the elementary school; however, her academic performance was left behind significantly.

### Metabolic dynamics of 6 functional networks

Among the 4 timepoints of PET studies during the course of PFS in this individual patient, PET imaging became an informative biomarker represented a snapshot of cerebrocerebellar function chronologically. The metabolic dynamics of 6 functional networks, including SMN, FPN, CON, OCC, DMN, and cerebellar network, of 4 timepoints (stages 1–4) are presented in Fig. 2. Among the 6 networks, the SMN, CON, and DMN were the 3 networks got earlier improvement and kept improving across all timepoints. A significant decrease of FPN and OCC at stage 2 comparing to stage 1 was noted. The cerebellar network (F) was relatively stable across all timepoints except a mild yet significant uptake increases from stage 2 to 3. In general, the metabolic uptake increased significantly from stages 1 and 2 to stages 3 and 4 in all functional networks, which correlated well with the course of neurological improvement in clinical observations (see also Fig. 1).

### Metabolic correlation coefficient matrices and connectivity networks

To further analyze the connectivity within each 120 anatomical structures, metabolic correlation coefficient matrices were generated comparing stages 2, 3, and 4 to stage 1, respectively (Fig. 3A). A clear increase of metabolic correlation with regional preference was observed during the recovery phase of PFS. Specifically, superior parietal lobule, occipital lobe, putamen, and vermis were the 4 regions enhanced prominently within stage 2-1 (Fig. 3A, left). When comparing stage 3 to stage 1, the amygdala, hippocampus, and parahippocampus, as well as cerebellum and vermis, were regions enhanced prominently (Fig. 3A, middle). When comparing stage 4 to stage 1, the temporal pole, amygdala, hippocampus and parahippocampus, thalamus, fusiform gyrus, olfactory, Rolandic operculum, and cerebellum and vermis were regions correlated strongly with nearly every other structure (Fig. 3A, right). Illustrative graphs demonstrated connectivity networks of cerebral (Fig. 3B) and cerebellum (Fig. 3C) in stage 2-1, stage 3-1, and stage 4-1, respectively. Collectively, a clear improvement of FC globally and regionally at both cerebrum and cerebellum has been observed through time which is compatible with findings in network analysis (Fig. 1).

### Continuous recovery of dentato-rubro-thalamo-cortical pathway

To test the hypothesis of pECP injury as a pathophysiology of PFS, we selected DN, RN, and thalamus as seed regions and analyzed their correlation with cortical areas (Fig. 4). Three correlation matrices between each stage: stage 2-1, stage 3-2, and stage 4-3 were generated to demonstrate the evolution of dentato-rubro-thalamo-cortical pathway, which is the major component of pECP, using an individualized connectivity network. Interestingly, the left DN recovered faster (stage 2-1) than right DN (stage 4-3), which correlated with the postoperative MRI in Fig. 1, showing a diffusion restriction region at right SCP. At stage 2-1, the left DN-RN-thalamic correlation developed. The left thalamo-cortical correlation was stronger than right side, and the earlier recovered thalamic projections were sending to 59.2% (45/76) of cortical regions except medial frontal, paracentral lobule, medial parietal, Rolandic operculum, lateral parietal, basal temporal, and occipital regions. At stage 3-2, no DN to RN correlation was detected, yet a continuous recovery of RN-thalamo-cortical projection was found. Besides, 97.4% (74/76) of cortical regions got connected to the thalamus. Of whom, 39.2% (29/74) of the connected power (correlation strength) were weaker in stage 3-2 than stage 2-1, suggesting an earlier recovery of these regions. On the contrary, 60.8% (45/74) of the correlation strength were stronger in stage 3-2 than stage 2-1, suggesting a continuous recovery of these regions. At stage 4-3, right DN-RN-thalamic correlation developed and 100% (76/76) of cortical regions correlated with thalamus of various degrees. To this stage, we identified regions with continuously improving of connected power to the thalamus, including olfactory, superior-medial frontal, Rolandic operculum, putamen, superior temporal pole, and fusiform gyri, which composed 15.8% (12/76) of cortical regions.

## Discussion

Since the first description of absence of speech, named as akinetic mutism, after removal of cerebellar tumor in a child by Daly and Love (1958), numerous studies have demonstrated syndromes centered on—but not limited to—mutism after posterior fossa surgery. (Hirsch et al. 1979; Kirk et al. 1995; Miller et al. 2011). Nevertheless, the terminology, such as using CMS, PFS, or others, to describe a collection of various degree of severity across different domain of neural functions remained inconsistent and confusing. A series of informative discussion highlighted the importance of standardizing identification and evaluation of the CMS/PFS (Thomale and Driever 2013). As has been elucidated by Gudrunardottir et al. who defined “CM,” referring to a delayed onset, limited duration and (usually) long-term linguistic sequelae, from “PFS,” of which CM as its main feature and encompasses motoric and neurobehavioral components (Gudrunardottir et al. 2011, 2011a, 2011b). In our work, we chose PFS instead of CMS to highlight the broad spectral symptomatology of our patient despite a recent consensus paper by an international group proposed “post-operative paediatric CMS” as a term to define pediatric patient group for the interests of future clinical and research work (Gudrunardottir et al. 2011). Furthermore, we proposed a grading system (Fig. 1D) modified from Gudrunardottir’s work (Gudrunardottir et al. 2011), which may serve as a reference to evaluate the severity of symptoms (Gudrunardottir et al. 2016). The combination of a grading on a chronologically recorded symptomatologies and a series of functional neural images provide a path for dissecting complex clinical syndromes from a network-based approach.

Like the story of the terminology in describing the PFS and the CMS, several macro-scale functional networks have been recognized and named according to their representative functions and anatomical locations. Nevertheless, the lack of consistency in universal network naming scheme, or taxonomy, may hinder the comparison or generalization among studies. To speak the same language, herein we refer our networks according to several seminal studies (Coste and Kleinschmidt 2016; Doucet et al. 2019) and elucidate the association of network dynamics (Fig. 2) with fluctuations of symptoms (Fig. 1). The FPN (Fig. 2B) is composed of frontal operculum, inferior parietal lobe, and angular gyrus, which may correspond to central executive network (CEN), while the CON (Fig. 2C) is composed of midcingulate region, hippocampus, amygdala, and basal ganglion, which may correspond to salience network (SN). Together with the DMN (Fig. 2E), these networks—the CEN, the DMN, and the SN—have been recognized as fundamental pathways for understanding higher cognitive function (Doucet et al. 2019; Uddin et al. 2019). On the contrary, the SMN (Fig. 2A) and the OCC (Fig. 2D) are 2 locally integrated and globally isolated networks, with their functions highly correlated with the anatomical morphology as well as low interindividual variability (Menon 2011). Finally, the cerebellar network (Fig. 2F) is composed of regions within cerebellar hemisphere and vermis (Power et al. 2011; Doucet et al. 2019).

We first analyzed improved clinical symptoms regarding different domains—motoric, linguistic, and neurobehavioral (Fig. 1C and D), and correlate the collection of symptoms to the dynamics of large-scale functional networks (Fig. 2). Comparing stage 2 to stage 1, she started to move limbs and grasp (motoric) and expressed temper by screaming and crying while sometimes laughing (neurobehavioral). There was neither eye opening nor verbal output yet (linguistic). In Fig. 2, no significant improvement for all networks could be identified from stage 1 to stage 2. Besides, the FC of the FPN and the OCC significantly decreased, which correlated with no verbal expression or eye opening. Comparing stage 3 to stage 2, she opened and rolled eyes. A rapid and significant improvement of linguistic and motoric symptoms was found. She could obey order, controlled her trunk and limbs although not very accurate for reaching and holding stuffs (motoric). She could count numbers and speak words although dysarthric (linguistic). Besides, her nasogastric tube was removed smoothly indicating a recovery of tongue and oropharyngeal functions. She still presented with emotional liability but able to control her temper. In Fig. 2, the FC of all 6 networks significantly improved from stage 2 to stage 3, which supported what has been observed clinically including trunk/limbs/orofacial movement (the SMN), eye-opening (the OCC), comprehend and speak meaningful words (the FPN; Marek and Dosenbach 2018) and order-obedience [the interaction of the DMN (Yakushev et al. 2013), the CON (Coste and Kleinschmidt 2016), and the FPN (Marek and Dosenbach 2018)]. Comparing stage 4 to stage 3, she could behave well despite having an irritable mood. She could walk although slightly wide-based and unsteadiness (cerebellar dysfunction). She had fewer talk than before; however, she could speak sentences and express her thoughts. In Fig. 2, the FC of all 5 cerebral networks improved even more significantly from stage 3 to stage 4, which supported a nearly full recovered functional status with minimal neurological sequelae related to cerebellar (pECP) dysfunction. Collectively, the dynamics of large-scale networks in Fig. 2 correlated well with the dynamics of symptomatologies of PFS in Fig. 1.

Several findings worth addressing. First, the cerebellar network is the least fluctuated network. Since the proposed pathomechanism of PFS is centered on pECP, which is the white matter bundle connecting to cerebral nucleus and cortices rather than a direct compromise on the intra-cerebellar connections, our findings may serve as evidence to support the influence of the cerebellum on the cerebral function via a widely connected cerebrocerebellar circuits (Buckner et al. 2011). Second, we found a trend of sooner recovery of the DMN and the CON comparing to the OCC, the SMN, and the FPN. Since there was no direct FC between cerebellum and primary visual and auditory cortices (Buckner et al. 2011), the order of recovery may represent the resilience of cerebral networks in respond to cerebellar damage, which may provide references to patients who suffered cerebellar insults present with complex symptoms in various domains clinically. Third, as is demonstrated in Fig. 4, we intended to demonstrate the FC among critical hubs: DN of cerebellum, RN of midbrain, thalamic nucleus, and their FC between various cortical regions (nodes). As mentioned previously, it appears that the dentato-rubro-thalamo-cortical pathway recovered earlier than the cortico-cortical projections. Such findings are in line with the modulating role of cerebellum on cerebral functions and may be explained by the delay of relay within these polysynaptic networks originated from dentate nuclei. Consequently, this may provide functional evidence to support the role pECP to the PFS identical to the structural evidence provided by DTI studies (Morris et al. 2009; Palesi et al. 2015).

The term “chronnectome” has been proposed to describe the dynamic connectivity of fMRI considering the time-varying properties to better characterize and understand brain functions (Calhoun et al. 2014). Of note, the FC varies dramatically across individuals highlights a group-level network analyses using static FC might mask subtle changes at individual level (Liao et al. 2017). Despite the utilization of chronnectomic fingerprinting on identifying individual higher cognitive functions (Liu et al. 2018), the fMRI could only provide a resolution of dynamics limited to seconds to minutes. For an evolving disease or specific collection of symptoms at the scale of days or even years, separate but serial functional images provide the opportunities to correlate the pathological mechanism underlying certain diseases (Watabe and Hatazawa 2019). Modified from the individual metabolic networks what we have verified on patients with cognitive impairment (Huang et al. 2020), it is possible that we focus on the association between dynamics of symptomatologies and brain networks in a single subject. The integration of network neuroscience using functional images to clinical neurology at an individual level offers valuable information regarding how brain functions and dysfunctions.

There are several limitations. First, this is a single subject analysis. The generalization of this approach to other diseases requires modification and validation. However, we do think this approach to longitudinally acquiring functional imaging provides opportunities to study the relationship of the parameters (network) to the answer (neurophysiological presentation) within each equation. Furthermore, the patient we present is a suitable candidate to demonstrate individualized network approach because she presented with full spectrum of CMS/PFS yet recovered nearly fully. For that, we could thoroughly discuss the relationships between symptomatologies and network connectivity. Second, we analyze the well-known networks of cerebral functional connectivity and the DTI-validated pECP using a network approach. In the pCMS, it is a cerebellar dysfunction affecting cerebral operation. We believe there are more complicated influences between cerebellum and cerebrum or even brain stem and cerebrum, which is not discussed in the current study. Third, the resolution of PET imaging is lower than functional MRI in terms of differentiating anatomical hubs and seeds. This may hinder a more detail study into rich deep connectivities such as networks involving basal ganglion and thalamus. The advantage of PET over fMRI is its stable signal-to-noise ratio and less bony artifact from basal skull, with the shortage including lower temporal resolution especially trying to detect short-term network variations. Therefore, the pros and cons of each functional imaging modality should be evaluated before study design.

## Conclusion

The progress of network neuroscience has been proved valuable for delineating how the CNS functions and its application on neurodegenerative and psychiatric diseases has shed light on understanding how these complicated disorder dysfunction. The postoperative CMS or PFS is a syndrome composed of various domains of cerebral functions originates from disruption of cerebellar output toward cerebrum. By providing a severity score for symptoms with a detailed clinical course and utilizing metabolic imaging, we demonstrate a dissection of the PFS in functional perspective chronologically. Since structural imaging such as DTI or volumetric MRI may not be sensitive enough to reflect clinical kinesis, functional imaging such as fMRI or PET provides drastic fluctuation of cerebrocerebellar functional networks, which serves as a perfect complement for what has been found from the structural MRI in understanding the PFS. Through a combinatorial approach with structural and functional imaging, knowledges developed in both fields could be template interchangeably. We believe such approach could be a powerful tool for researchers studying neurology and neuroscience.

## Supplementary Material

Supplementary_tgac008

## References

[ref1] Avula S . Radiology of post-operative paediatric cerebellar mutism syndrome. Childs Nerv Syst. 2020:36(6):1187–1195. 31183530 10.1007/s00381-019-04224-x

[ref2] Becker BJ . Synthesizing standardized mean-change measures. Br J Math Stat Psychol. 1988:41(2):257–278.

[ref3] Buckner RL, Krienen FM, Castellanos A, Diaz JC, Yeo BT. The organization of the human cerebellum estimated by intrinsic functional connectivity. J Neurophysiol. 2011:106(5):2322–2345. 21795627 10.1152/jn.00339.2011 PMC3214121

[ref4] Calhoun VD, Miller R, Pearlson G, Adalı T. The chronnectome: time-varying connectivity networks as the next frontier in fMRI data discovery. Neuron. 2014:84(2):262–274. 25374354 10.1016/j.neuron.2014.10.015 PMC4372723

[ref5] Catsman-Berrevoets CE, Aarsen FK. The spectrum of neurobehavioural deficits in the posterior fossa syndrome in children after cerebellar tumour surgery. Cortex. 2010:46(7):933–946. 20116053 10.1016/j.cortex.2009.10.007

[ref6] Catsman-Berrevoets C, Patay Z. Cerebellar mutism syndrome. Handb Clin Neurol. 2018:155:273–288. 29891065 10.1016/B978-0-444-64189-2.00018-4

[ref7] Chen D, Lu J, Zhou H, Jiang J, Wu P, Guo Q, Ge J, Zhang H, Shi K, Zuo C. Glucose metabolic brain network differences between Chinese patients with Lewy body dementia and healthy control. Behav Neurol. 2018:2018:8420658. 29854020 10.1155/2018/8420658 PMC5964431

[ref8] Coste CP, Kleinschmidt A. Cingulo-opercular network activity maintains alertness. NeuroImage. 2016:128:264–272. 26801604 10.1016/j.neuroimage.2016.01.026

[ref9] Daly DD, Love JG. Akinetic mutism. Neurology. 1958:8(3):238. 13517492 10.1212/wnl.8.3.238

[ref10] Doucet G, Lee W, Frangou S. Evaluation of the spatial variability in the major resting-state networks across human brain functional atlases. Hum Brain Mapp. 2019:40(15):4577–4587. 31322303 10.1002/hbm.24722 PMC6771873

[ref11] Erşahin Y, Mutluer S, Cağli S, Duman Y. Cerebellar mutism: report of seven cases and review of the literature. Neurosurgery. 1996:38(1):60–65 discussion 66. 8747952 10.1097/00006123-199601000-00015

[ref12] Gudrunardottir T, Sehested A, Juhler M, Grill J, Schmiegelow K. Cerebellar mutism: definitions, classification and grading of symptoms. Childs Nerv Syst. 2011:27(9):1361–1363. 21732118 10.1007/s00381-011-1509-7

[ref13] Gudrunardottir T, Sehested A, Juhler M, Schmiegelow K. Cerebellar mutism: incidence, risk factors and prognosis. Childs Nerv Syst. 2011a:27(4):513–514 author reply 515. 21318616 10.1007/s00381-010-1383-8

[ref14] Gudrunardottir T, Sehested A, Juhler M, Schmiegelow K. Cerebellar mutism: review of the literature. Childs Nerv Syst. 2011b:27(3):355–363. 21061011 10.1007/s00381-010-1328-2

[ref15] Gudrunardottir T, Morgan AT, Lux AL, Walker DA, Walsh KS, Wells EM, Wisoff JH, Juhler M, Schmahmann JD, Keating RF, et al. Consensus paper on post-operative pediatric cerebellar mutism syndrome: the Iceland Delphi results. Childs Nerv Syst. 2016:32(7):1195–1203. 27142103 10.1007/s00381-016-3093-3

[ref16] He Y, Wang J, Wang L, Chen ZJ, Yan C, Yang H, Tang H, Zhu C, Gong Q, Zang Y, et al. Uncovering intrinsic modular organization of spontaneous brain activity in humans. PLoS One. 2009:4(4):e5226. 19381298 10.1371/journal.pone.0005226 PMC2668183

[ref17] Hirsch JF, Renier D, Czernichow P, Benveniste L, Pierre-Kahn A. Medulloblastoma in childhood. Survival and functional results. Acta Neurochir. 1979:48(1–2):1–15. 495234 10.1007/BF01406016

[ref18] Huang SY, Hsu JL, Lin KJ, Hsiao IT. A novel individual metabolic brain network for 18F-FDG PET imaging. Front Neurosci. 2020:14:344. 32477042 10.3389/fnins.2020.00344 PMC7235322

[ref19] Imai M, Tanaka M, Sakata M, Wagatsuma K, Tago T, Toyohara J, Sengoku R, Nishina Y, Kanemaru K, Ishibashi K, et al. Metabolic network topology of Alzheimer’s disease and dementia with Lewy bodies generated using fluorodeoxyglucose positron emission tomography. J Alzheimers Dis. 2020:73(1):197–207. 31771066 10.3233/JAD-190843 PMC7029362

[ref20] Kim HY . Statistical notes for clinical researchers: effect size. Restor Dent Endod. 2015:40(4):328–331. 26587420 10.5395/rde.2015.40.4.328 PMC4650530

[ref21] Kirk EA, Howard VC, Scott CA. Description of posterior fossa syndrome in children after posterior fossa brain tumor surgery. J Pediatr Oncol Nurs. 1995:12(4):181–187. 7495523 10.1177/104345429501200402

[ref22] Koh S, Turkel SB, Baram TZ. Cerebellar mutism in children: report of six cases and potential mechanisms. Pediatr Neurol. 1997:16(3):218–219. 9165512 10.1016/s0887-8994(97)00018-0 PMC3399684

[ref23] Leiner HC, Leiner AL, Dow RS. Cerebro-cerebellar learning loops in apes and humans. Ital J Neurol Sci. 1987:8(5):425–436. 3323123 10.1007/BF02334599

[ref24] Liao X, Cao M, Xia M, He Y. Individual differences and time-varying features of modular brain architecture. NeuroImage. 2017:152:94–107. 28242315 10.1016/j.neuroimage.2017.02.066

[ref25] Liu J, Liao X, Xia M, He Y. Chronnectome fingerprinting: identifying individuals and predicting higher cognitive functions using dynamic brain connectivity patterns. Hum Brain Mapp. 2018:39(2):902–915. 29143409 10.1002/hbm.23890 PMC6866558

[ref26] Lucignani G, Paganelli G, Bombardieri E. The use of standardized uptake values for assessing FDG uptake with PET in oncology: a clinical perspective. Nucl Med Commun. 2004:25(7):651–656. 15208491 10.1097/01.mnm.0000134329.30912.49

[ref27] Marek S, Dosenbach NUF. The frontoparietal network: function, electrophysiology, and importance of individual precision mapping. Dialogues Clin Neurosci. 2018:20(2):133–140. 30250390 10.31887/DCNS.2018.20.2/smarek PMC6136121

[ref28] Mariën P, De Smet HJ, Wijgerde E, Verhoeven J, Crols R, Deyn D. Posterior fossa syndrome in adults: a new case and comprehensive survey of the literature. Cortex. 2013:49(1):284–300. 21855865 10.1016/j.cortex.2011.06.018

[ref29] Menon V . Large-scale brain networks and psychopathology: a unifying triple network model. Trends Cogn Sci. 2011:15(10):483–506. 21908230 10.1016/j.tics.2011.08.003

[ref30] Miller NG, Reddick WE, Kocak M, Glass JO, Löbel U, Morris B, Gajjar A, Patay Z. Cerebellocerebral diaschisis is the likely mechanism of postsurgical posterior fossa syndrome in pediatric patients with midline cerebellar tumors. AJNR Am J Neuroradiol. 2010:31(2):288–294. 19797787 10.3174/ajnr.A1821 PMC3568945

[ref31] Miller A, Pratt H, Schiffer RB. Pseudobulbar affect: the spectrum of clinical presentations, etiologies and treatments. Expert Rev Neurother. 2011:11(7):1077–1088. 21539437 10.1586/ern.11.68

[ref32] Morris SB, DeShon RP. Combining effect size estimates in meta-analysis with repeated measures and independent-groups designs. Psychol Methods. 2002:7(1):105–125. 11928886 10.1037/1082-989x.7.1.105

[ref33] Morris EB, Phillips NS, Laningham FH, Patay Z, Gajjar A, Wallace D, Boop F, Sanford R, Ness KK, Ogg RJ. Proximal dentatothalamocortical tract involvement in posterior fossa syndrome. Brain J Neurol. 2009:132(Pt 11):3087–3095. 10.1093/brain/awp241 PMC2781745 19805491

[ref34] Palesi F, Tournier JD, Calamante F, Muhlert N, Castellazzi G, Chard D, D'Angelo E, Wheeler-Kingshott CA. Contralateral cerebello-thalamo-cortical pathways with prominent involvement of associative areas in humans in vivo. Brain Struct Funct. 2015:220(6):3369–3384. 25134682 10.1007/s00429-014-0861-2 PMC4575696

[ref35] Power JD, Cohen AL, Nelson SM, Wig GS, Barnes KA, Church JA, Vogel AC, Laumann TO, Miezin FM, Schlaggar BL, et al. Functional network organization of the human brain. Neuron. 2011:72(4):665–678. 22099467 10.1016/j.neuron.2011.09.006 PMC3222858

[ref36] Riva D . The cerebellar contribution to language and sequential functions: evidence from a child with cerebellitis. Cortex. 1998:34(2):279–287. 9606593 10.1016/s0010-9452(08)70755-x

[ref37] Riva D, Giorgi C. The cerebellum contributes to higher functions during development: evidence from a series of children surgically treated for posterior fossa tumours. Brain. 2000:123(Pt 5):1051–1061. 10775549 10.1093/brain/123.5.1051

[ref38] Robertson PL, Muraszko KM, Holmes EJ, Sposto R, Packer RJ, Gajjar A, Dias MS, Allen JC. Incidence and severity of postoperative cerebellar mutism syndrome in children with medulloblastoma: a prospective study by the Children's Oncology Group. J Neurosurg. 2006:105(6 Suppl):444–451. 17184075 10.3171/ped.2006.105.6.444

[ref39] Sagiuchi T, Ishii K, Aoki Y, Kan S, Utsuki S, Tanaka R, Fujii K, Hayakawa K. Bilateral crossed cerebello-cerebral diaschisis and mutism after surgery for cerebellar medulloblastoma. Ann Nucl Med. 2001:15(2):157–160. 11448076 10.1007/BF02988609

[ref40] Sanabria-Diaz G, Martínez-Montes E, Melie-Garcia L. Glucose metabolism during resting state reveals abnormal brain networks organization in the Alzheimer’s disease and mild cognitive impairment. PLoS One. 2013:8(7):e68860. 23894356 10.1371/journal.pone.0068860 PMC3720883

[ref41] Schmahmann JD . The cerebellum and cognition. Neurosci Lett. 2019:688:62–75. 29997061 10.1016/j.neulet.2018.07.005

[ref42] Sergeant A, Kameda-Smith MM, Manoranjan B, Karmur B, Duckworth J, Petrelli T, Savage K, Ajani O, Yarascavitch B, Samaan MC, et al. Analysis of surgical and MRI factors associated with cerebellar mutism. J Neuro-Oncol. 2017:133(3):539–552. 10.1007/s11060-017-2462-4 28527006

[ref43] Thomale UW, Driever PH. Inconsistent terminology for cerebellar mutism. Childs Nerv Syst. 2013:29(5):717–718. 23503612 10.1007/s00381-013-2074-z

[ref44] Toescu SM, Hettige S, Phipps K, Smith RJP, Haffenden V, Clark C, Hayward R, Mankad K, Aquilina K. Post-operative paediatric cerebellar mutism syndrome: time to move beyond structural MRI. Childs Nerv Syst. 2018:34(11):2249–2257. 29926177 10.1007/s00381-018-3867-x PMC6208673

[ref45] Trobe JD . The human brain. An introduction to its functional anatomy, 6th edition. J Neuroophthalmol. 2010:30(1):107.

[ref46] Tzourio-Mazoyer N, Landeau B, Papathanassiou D, Crivello F, Etard O, Delcroix N, Mazoyer B, Joliot M. Automated anatomical labeling of activations in SPM using a macroscopic anatomical parcellation of the MNI MRI single-subject brain. NeuroImage. 2002:15(1):273–289. 11771995 10.1006/nimg.2001.0978

[ref47] Uddin LQ, Yeo BTT, Spreng RN. Towards a universal taxonomy of macro-scale functional human brain networks. Brain Topogr. 2019:32(6):926–942. 31707621 10.1007/s10548-019-00744-6 PMC7325607

[ref48] Watabe T, Hatazawa J. Evaluation of functional connectivity in the brain using positron emission tomography: a mini-review. Front Neurosci. 2019:13:775. 31402852 10.3389/fnins.2019.00775 PMC6676772

[ref49] Yakushev I, Chételat G, Fischer FU, Landeau B, Bastin C, Scheurich A, Perrotin A, Bahri MA, Drzezga A, Eustache F, et al. Metabolic and structural connectivity within the default mode network relates to working memory performance in young healthy adults. NeuroImage. 2013:79:184–190. 23631988 10.1016/j.neuroimage.2013.04.069

