# Supplementary material for: Individual cerebrocerebellar functional network analysis decoding symptomatologic dynamics of postoperative cerebellar mutism syndrome
Source: Cereb Cortex Commun. 2022 Feb 11;3(1):tgac008. doi: 10.1093/texcom/tgac008 (PMC8914218; doi:10.1093/texcom/tgac008)
Supplement: Supplementary_tgac008 [file supplementary_tgac008.pdf]

Title: Individual Cerebrocerebellar Functional Network Analysis for Decoding the Symptomatological Dynamics of Posterior Fossa Syndrome

Supplementary Materials

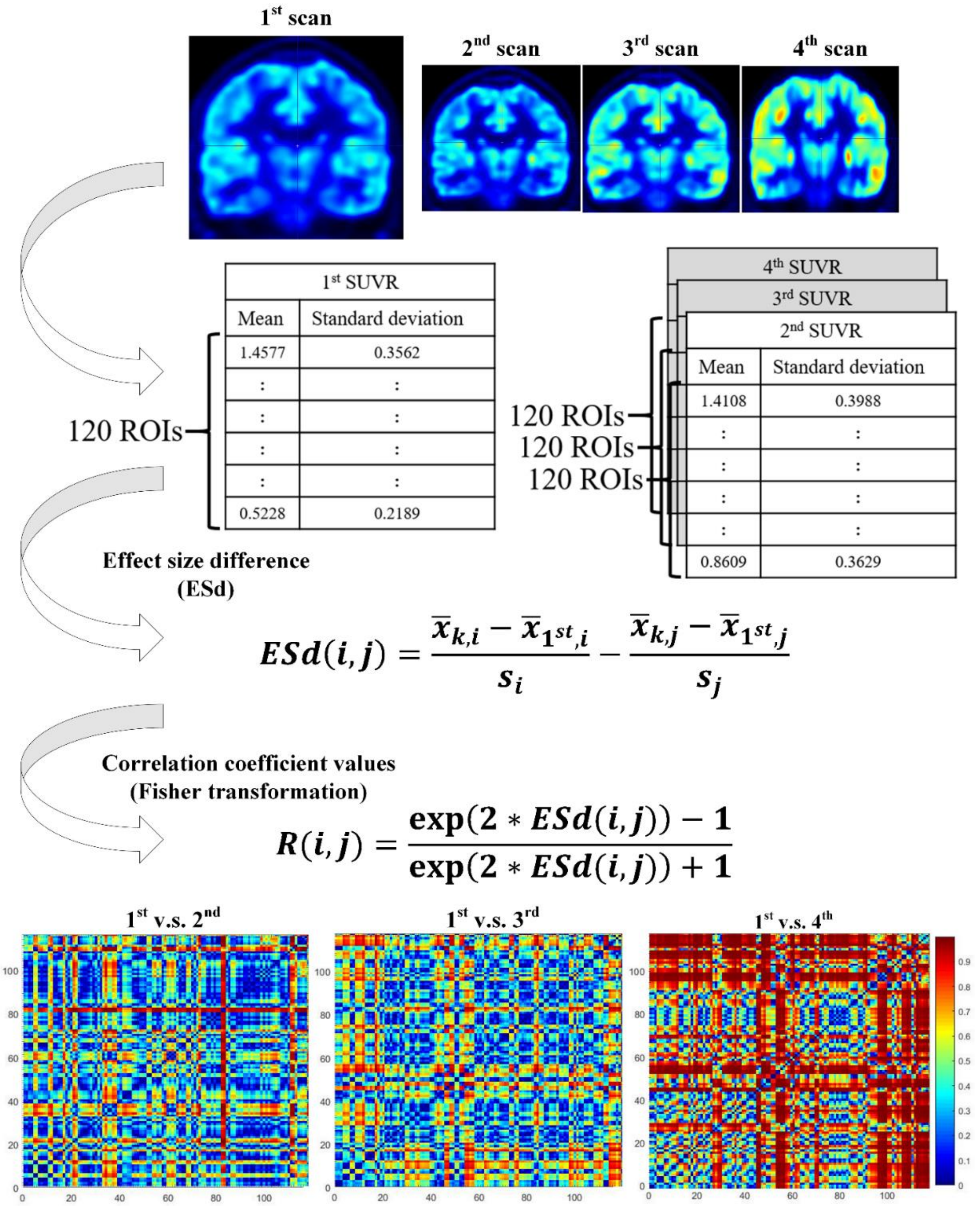

Figure S1. Algorithm of the individual brain network analysis. (A) Each scan normalized at a different time point. The correlation coefficient values between each

pair of nodes were the calculated after standardizing to 120 regions of interest. (B) A correlation coefficient matrix was generated for stages 2 and 1, stages 3 and 1, and stages 4 and 1, indicating that brain connectivity progressively and continuously recovered.

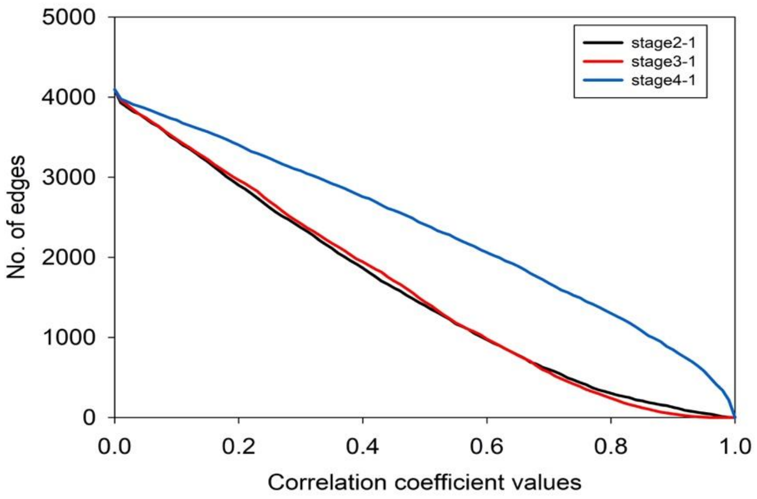

Figure S2. The resulting number of edges in the inter-hemispheric connectivity network for each brain network (stage2-1, stage3-1, stage4-1). One can observe a significant reduction of inter-hemispheric connections in stage2-1 and stage3-1.

Figure 1

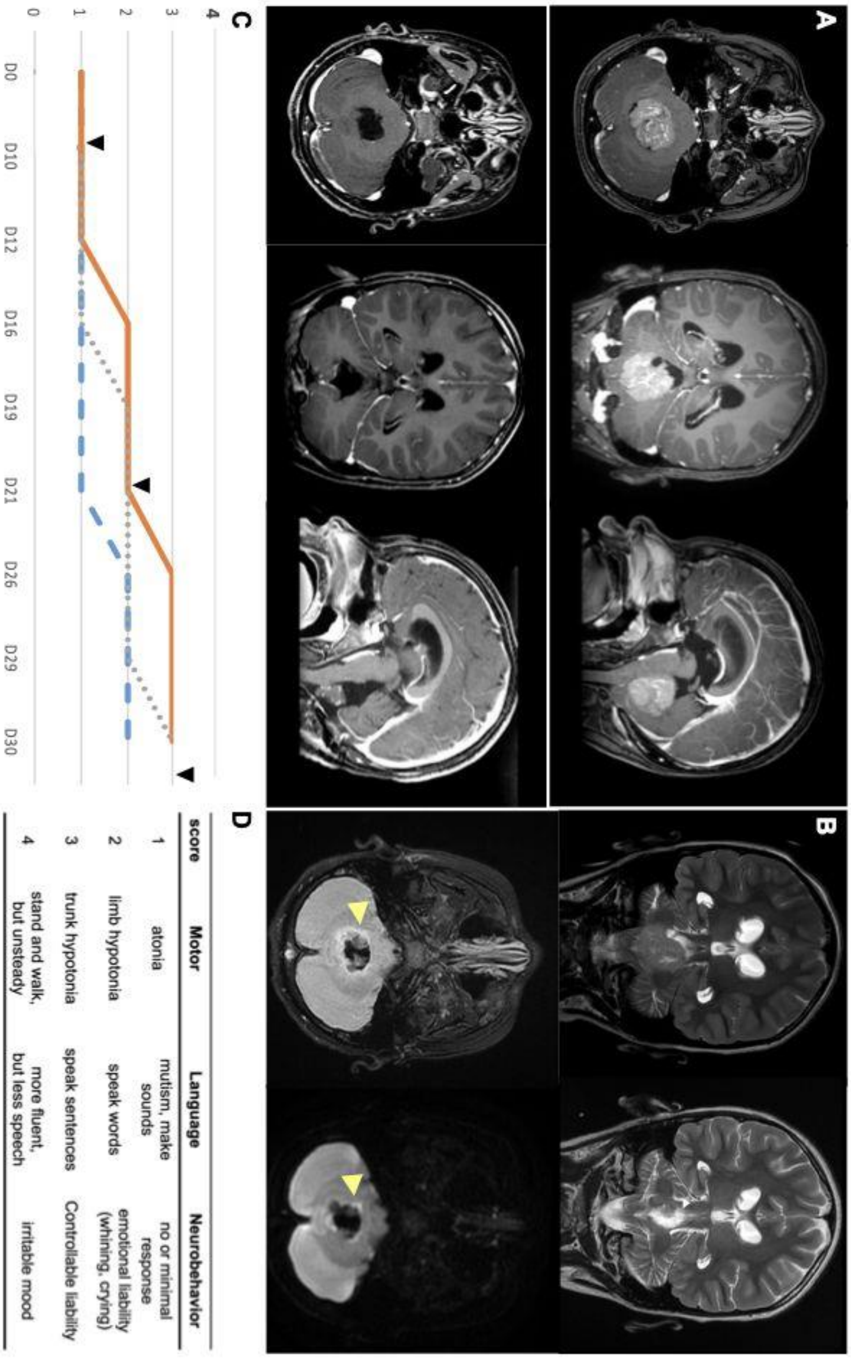

score	Motor	Language	Neurobehavior
1	atonia	mutism, make sounds	no or minimal response
2	limb hypotonia	speak words	emotional liability (whining, crying)
3	trunk hypotonia	speak sentences	Controllable liability
4	stand and walk, but unsteady	more fluent, but less speech	irritable mood

Figure 3A (Left)

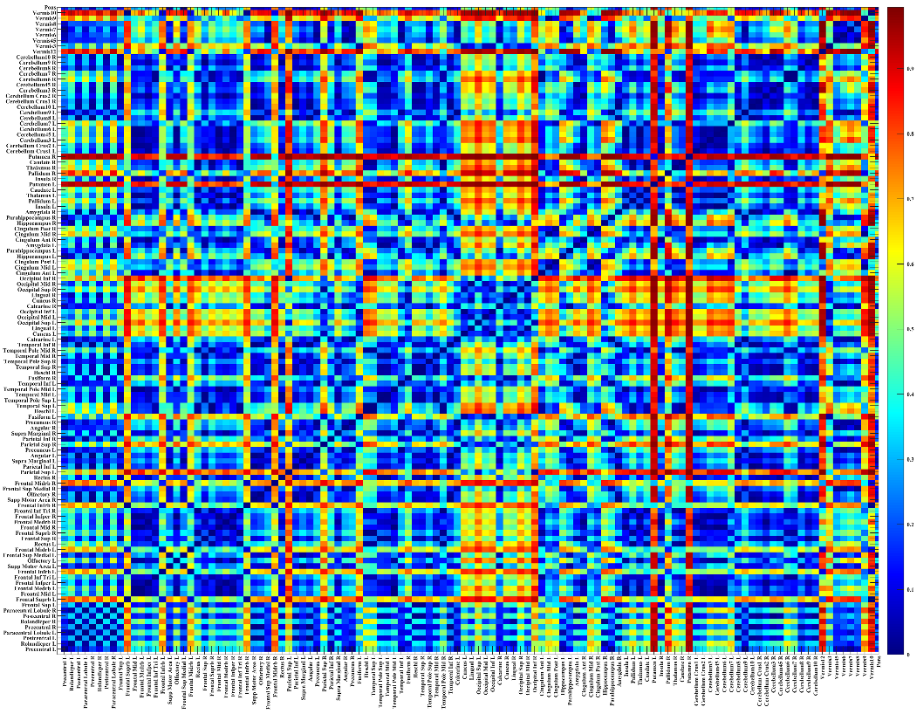

Figure 3A (Middle)

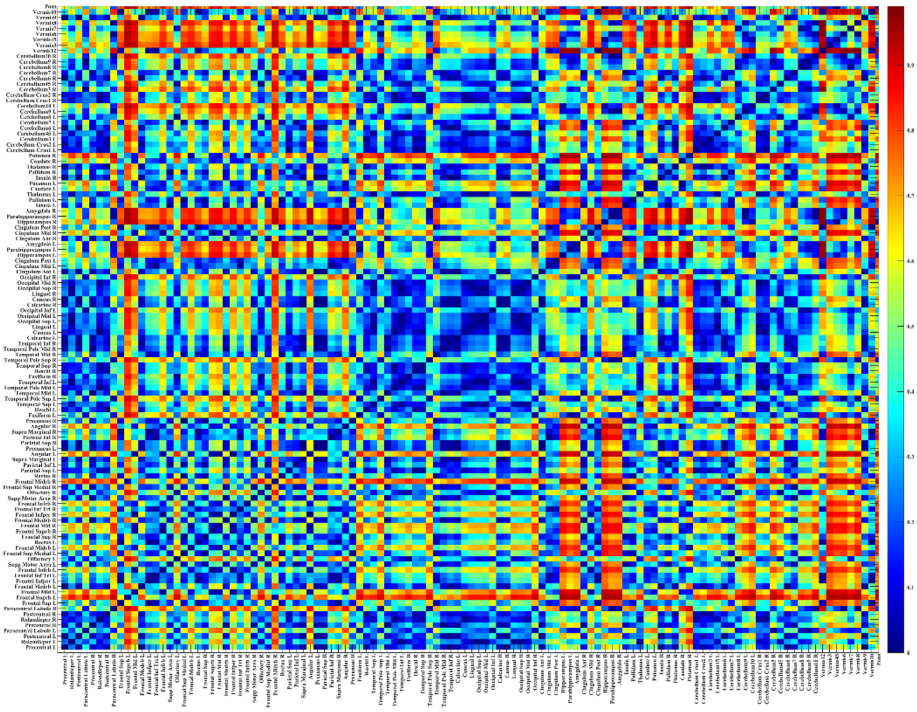

Figure 3A (Right)

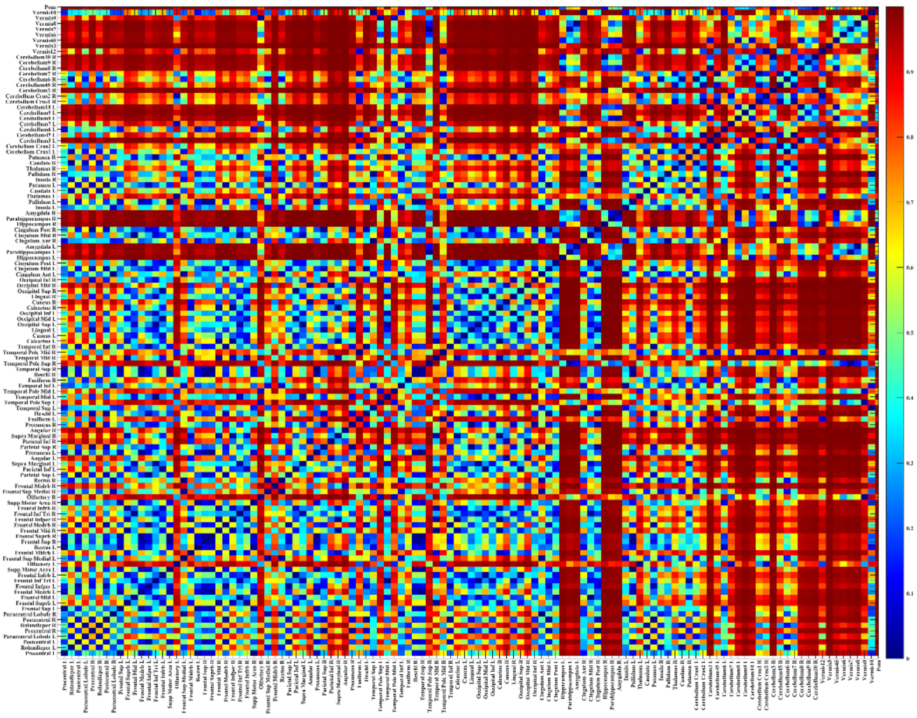

Figure 3B

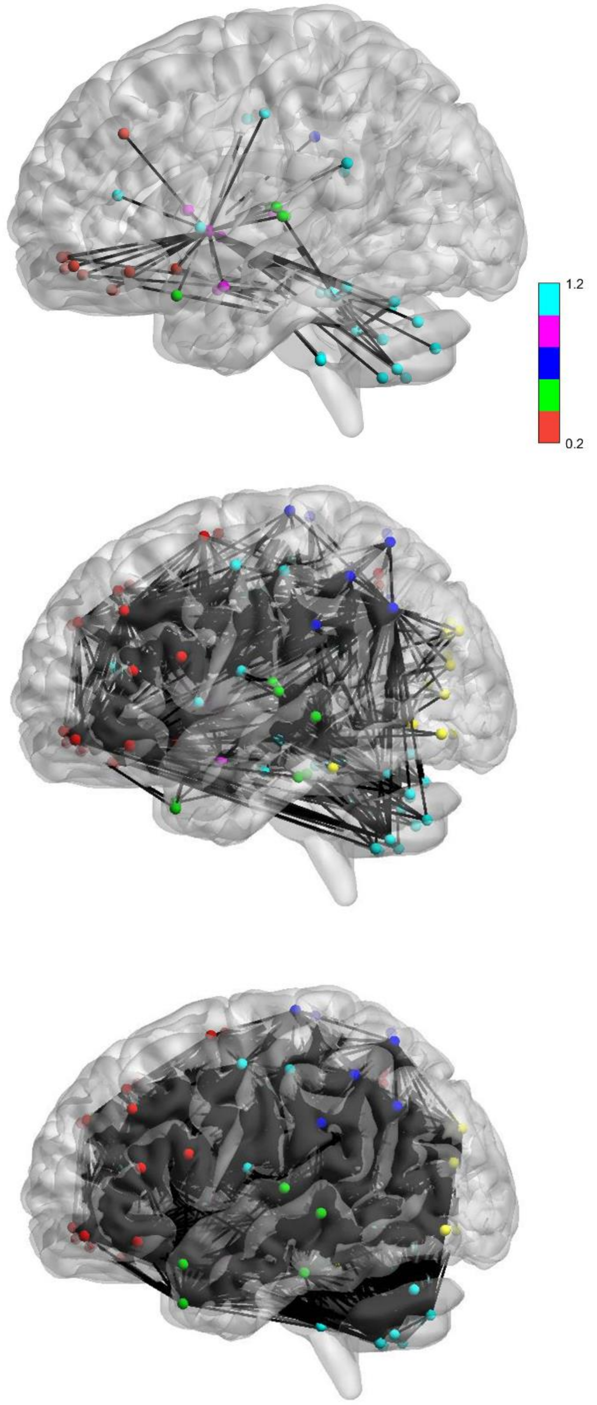

Figure 3C

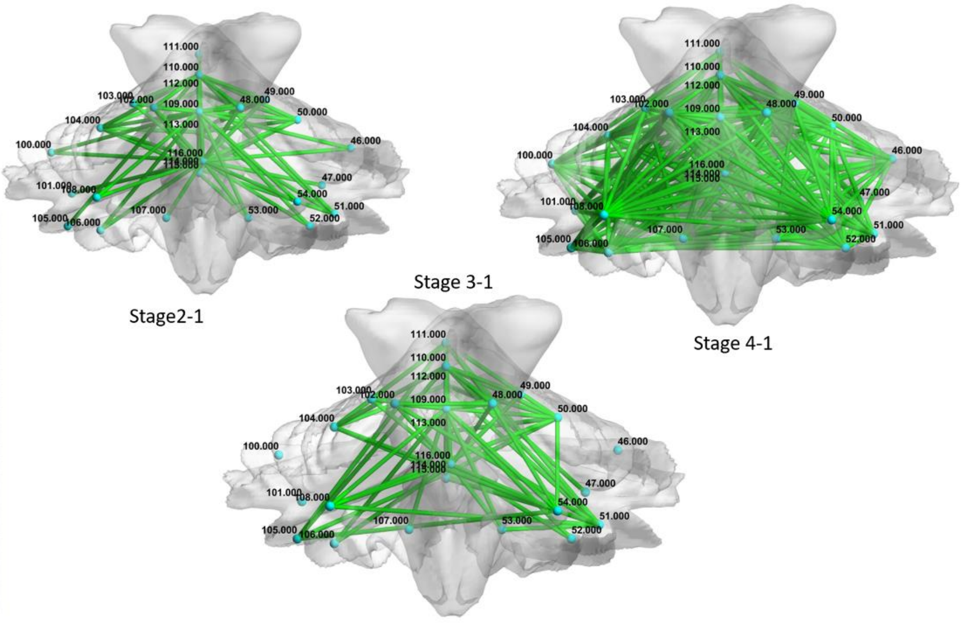

46	Cerebellum_Crus1 L
47	Cerebellum_Crus2 L
48	Cerebellum3 L
49	Cerebellum45 L
50	Cerebellum6 L
51	Cerebellum7 L
52	Cerebellum8 L
53	Cerebellum9 L
54	Cerebellum10 L
100	Cerebellum_Crus1 R
101	Cerebellum_Crus2 R
102	Cerebellum3 R
103	Cerebellum45 R
104	Cerebellum6 R
105	Cerebellum7 R
106	Cerebellum8 R
107	Cerebellum9 R
108	Cerebellum10 R
109	Vermis12
110	Vermis3
111	Vermis45
112	Vermis6
113	Vermis7
114	Vermis8
115	Vermis9
116	Vermis10
